# Molecular characterization of *Pseudomonas* from *Agaricus bisporus* caps reveal novel blotch pathogens in Western Europe

**DOI:** 10.1186/s12864-020-06905-3

**Published:** 2020-07-22

**Authors:** Tanvi Taparia, Marjon Krijger, Edward Haynes, John G. Elphinstone, Ralph Noble, Jan van der Wolf

**Affiliations:** 1 Biointeractions and Plant Health, Wageningen University and Research, Wageningen, Netherlands; 2 Department of Microbial Ecology, Netherlands Institute of Ecology, Wageningen, Netherlands; 3 Department of Plant Protection, Fera Science Limited, York, UK; 4 Pershore College, Warwickshire College Group, Worcestershire, UK

**Keywords:** Multilocus sequence alignment, Average nucleotide identity, In-silico DNA DNA hybridization, *“Pseudomonas gingeri”*, *Pseudomonas* sp. *NC02*, *Serratia* spp., *Pseudomonas salomonii*, *Pseudomonas yamanorum*, *Pseudomonas edaphica*, Bacterial blotch, Pathogenicity, Pot test, Cap test

## Abstract

**Background:**

Bacterial blotch is a group of economically important diseases affecting the cultivation of common button mushroom, *Agaricus bisporus*. Despite being studied for more than a century, the identity and nomenclature of blotch-causing *Pseudomonas* species is still unclear. This study aims to molecularly characterize the phylogenetic and phenotypic diversity of blotch pathogens in Western Europe.

**Methods:**

In this study, blotched mushrooms were sampled from farms across the Netherlands, United Kingdom and Belgium. Bacteria were isolated from symptomatic cap tissue and tested in pathogenicity assays on fresh caps and in pots. Whole genome sequences of pathogenic and non-pathogenic isolates were used to establish phylogeny via multi-locus sequence alignment (MLSA), average nucleotide identity (ANI) and in-silico DNA:DNA hybridization (DDH) analyses.

**Results:**

The known pathogens “*Pseudomonas gingeri*”, *P. tolaasii*, “*P. reactans*” and *P. costantinii* were recovered from blotched mushroom caps. Seven novel pathogens were also identified, namely, *P. yamanorum, P. edaphica, P. salomonii* and strains that clustered with *Pseudomonas* sp. *NC02* in one genomic species*,* and three non-pseudomonads, i.e. *Serratia liquefaciens, S. proteamaculans* and a *Pantoea* sp. Insights on the pathogenicity and symptom severity of these blotch pathogens were also generated.

**Conclusion:**

A detailed overview of genetic and regional diversity and the virulence of blotch pathogens in Western Europe, was obtained via the phylogenetic and phenotypic analyses. This information has implications in the study of symptomatic disease expression, development of diagnostic tools and design of localized strategies for disease management.

## Background

Commercial button mushroom cultivation relies heavily on the dynamic interactions between *Agaricus bisporus* and the casing soil microflora [1]. The transformation of vegetative mycelium into a fruiting body is initiated by beneficial microbes in the casing soil [2–4]. However, the casing soil also introduces pathogenic microbes into mushroom farms, including blotch causing *Pseudomonas* species [5–7]. The humid and mesophilic conditions required for mushroom production are highly conducive to the enrichment and spread of such pathogens. Reliable identification and early detection is thus essential to avoid disease outbreaks.

The genus *Pseudomonas* is one of the most complex genera of Gram negative bacteria due to its large size of 114 species [8]. They form a major proportion (~ 40%) of the total culturable bacteria obtained from casing soil in mushroom farms [9]. While some of these are essential for stimulating the pinning of button mushrooms (e.g. *P. putida*) [1, 10], others are detrimental to crop health (e.g. *P. tolaasii*) [11, 12].

Bacterial blotch is a group of diseases that result in discolouration and disfiguration of mushroom caps in *A. bisporus*, due to fungal production of phenols and tyrosinases [13]. This reduces the total marketable crop due to compromised aesthetic value, lowers the shelf-life post-harvest, and lessens the overall yields due to pin death. These aspects of bacterial blotch jointly lead to severe economic losses [14–16]. Various *Pseudomonas* species are the main causative agents of blotch diseases on mushroom caps [7].

*P. tolaasii* causes small sunken dark brown spots or lesions on the mushroom cap that are referred to as “brown blotch” [11, 12]. *“P. reactans”* is known to cause varying discoloration from dark to light, accompanied by a surface depression [17] and *“P. gingeri”* produces ginger coloured discolorations that are more spread out on the cap surface, called “ginger blotch” [18]. Both of these species have not been formally described. *P. agarici* is the causative agent of “drippy gill” on *A. bisporus* and “yellow blotch” on oyster mushrooms (*Pleurotus* spp.), where it leads to relatively pale discolorations [19]. Global reports also indicate the role of other *Pseudomonas*, such as *P. costantinii, P. fluorescens* and *P. marginalis* in bacterial blotch diseases, with large phenotypic variation within and across species [20].

Blotch pathogens can be considered as endemic to the casing soil, an artificially prepared growth media composed of peat and lime, that is added on top of the compost [5, 21]. They have been found on healthy crops at similar densities to that of diseased crops [9]. It has thus been suggested that not just the pathogen density, but the composition of *Pseudomonas* species in the casing soil, especially the relative abundance of beneficial and disease-causing species, can be an important indicator for disease outbreaks [9]. A deeper understanding of the beneficials and pathogens within the genus is hence necessary.

Bacterial blotch has been studied for over a century [22, 23], despite which the identity and nomenclature of blotch-causing *Pseudomonas* is still unclear. Recent molecular investigations clarify the taxonomy of some blotch pathogens [24–27]. However, knowledge on the identity, diversity and pathogenicity of mushroom-associated *Pseudomonas* species at the regional scale is still lacking. This information is instrumental for the development of localized strategies for diagnostics, disease control and breeding of varieties.

In this study, we isolated *Pseudomonas* from blotched mushrooms on farms in the Netherlands, United Kingdom and Belgium. We performed whole genome sequence analyses of pathogenic isolates to develop a deeper understanding of the genetic diversity among the pathogens in Western Europe. Some non-pathogenic isolates were also included in the study. The molecular characterization of these isolates provides insights into the phylogenetic relationships between beneficial and blotch-causing *Pseudomonas* species commonly associated with the button mushroom, *A. bisporus.*

## Methods

### Bacterial isolations

Blotched mushrooms were sampled from commercial farms in the Netherlands, United Kingdom and Belgium for isolation of blotch-causing *Pseudomonas* species. Biopsies from symptomatic tissue of the cap surface (2 cm^2^ area) were made in sterilized Ringer’s solution [28], and homogenized in a polyethylene bag (Bioreba, Switzerland). The extract from each biopsy was dilution plated on King’s B medium [29]. After incubation at 25 °C for 48 h, single colonies were picked and re-plated. In total, 161 single colonies of suspected *Pseudomonas* spp., that were fluorescent under UV light (365 nm), were plated to pure cultures by re-streaking on King’s B medium. One isolation was also made from a healthy mushroom that did not display visible blotch symptoms. A list of bacterial isolates is presented as Additional file 1.

### Pathogenicity assays

All isolates were tested in an in vitro assay to check their pathogenicity. Bacterial strains were cultured in King’s B medium [29] at 25 °C for 24 h, and tested in the bioassay. Similarly sized cap surfaces (4–5 cm in diameter) of healthy mushrooms were placed on damp filter paper and inoculated with 20 μl of aqueous bacterial suspension of 10^6^ colony-forming units (cfu) per ml from the isolate, and tested in replicates of three. The mushrooms were incubated under high humidity conditions for 72 h at 20 °C. The development of blotch symptoms on the cap surface was observed visually and photographed. The isolates were scored, between 0 and 3, with the ascending numbers referring to non-pathogenic, mild, moderate, and severe symptoms for bacterial blotch [25]. Negative controls consisted of uninoculated mushroom caps and sterile water inoculated mushroom caps.

A selection of isolates were re-tested in pot assays. Mushrooms were grown in plastic pots (230 mm diameter × 220 mm depth) containing 4 kg of Phase III compost, spawn-run with the most commonly cultivated mushroom strain, Sylvan A15. The pots were cased with 1.3 L of casing soil (moist mixture of peat and sugar beet lime). The pots were watered with sterile water and incubated at 25 °C for 7 days. The room was then ventilated and the air temperature reduced to 18 °C and the relative humidity was maintained at 91–93%, until the end of the cultivation cycle. After 5 days, the casing soil in each pot was inoculated with 50 ml of aqueous bacterial suspension of 10^7^ cfu/ml. The development of blotch symptoms on the mushrooms was recorded over two flushes and scored as above. The type of blotch symptoms (brown, ginger or others) was recorded and photographed. Negative controls consisted of casing soil inoculated with sterile water.

### DNA extraction and sequencing

For NL and BE isolates, 250 mg of bacterial slime was picked from a pure culture on an agar plate, and used as starting material. Total DNA was extracted using Wizard Magnetic DNA Purification System for Food (Promega, United States) according to the manufacturer’s protocol, including the DNase-free RNAse treatment. Library construction was performed using Illumina Truseq Nano (Illumina, United States) with 1 μg of bacterial DNA. 125 bp paired-end sequencing of the DNA libraries was done using HiSeq2500 (Illumina, United States).

For UK isolates, a single colony was picked from each agar plate and extracted using the Qiagen DNeasy Blood and tissue kit following the manufacturer’s protocol. The DNA was quantified fluorometrically using a Quant-iT PicoGreen dsDNA Assay Kit (Thermo Fisher Scientific, United States) on the Infinite M200 PRO (Tecon, Switzerland) and then stored at − 80 °C for downstream processing. Library construction was performed using Illumina Nextera XT library preparation kit (Illumina, United States) with 0.8 ng of bacterial DNA. Sequencing of the DNA libraries was performed on the MiSeq (Illumina, United States) using the V3 Reagent Kit, generating 300 bp paired-end sequences.

The combined dataset included 68 newly generated genome sequences from bacteria isolated from symptomatic cap tissue, 30 sequences of mushroom-associated *Pseudomonas* species from a previous sampling [25] and 15 sequences of related strains obtained from NCBI (https://www.ncbi.nlm.nih.gov/). Quality control was performed on the raw reads prior to read mapping using CLC Genomics Workbench (QIAGEN, Germany). Adapter sequences were removed from the raw reads. Bases with Phred quality scores less than 20 based on a modified-Mott algorithm were trimmed. Raw reads greater than 1000 bp and less than 45 bp were discarded. Reads were trimmed to a final length of 125 bp (NL and BE isolates) and 300 bp (UK isolates). Trimmed reads were mapped to the reference genomes without masking. Non-specific matches were randomly mapped.

### Determination of prokaryotic taxonomy

Multi-Locus Sequence Alignment (MLSA) with trimmed coding sequences of eleven barcoding genes from 13 reference strains were used to establish phylogeny between the isolates [30]. Housekeeping genes were chosen as phylogenetic molecular markers based on several criteria. The genes had a single copy number, they code universally for ubiquitous proteins with housekeeping functions, are likely recalcitrant to the effects of horizontal gene transfer, are long enough (> 900 bp) to contain sufficient information, and can predict whole-genome relationships [31]. Trimmed reads were mapped to the concatenated sequences of individual barcoding genes from multiple reference strains, using Map Reads to Reference 1.6 with a similarity and length fraction of 0.9 (CLC Genomics Workbench 11.0.2). Consensus DNA sequences were extracted from the mapping, and used for making phylogenetic trees with maximum likelihood and maximum parsimony methods [32]. Graphics from phylogenetic trees were made in RStudio [33] using package ggtree [34].

Genome assemblies were performed on the trimmed reads using De Novo Assembly 1.4 with a minimum contig length of 200 bp (CLC Genomics Workbench 11.0.2). Legacy BLAST [35] based Average Nucleotide Identity (ANI) analysis [36] was performed on the contig sequences from the assembled genomes using pyani 0.2.9 [37]. Similarity value of 95% was used as cut-off threshold for identification of a unique taxonomic group. The similarity values were used for phylogenetic analyses and to create graphics in RStudio [33]. To clarify the taxonomy of isolates that did not cluster together with any of the reference strains in the ANI or the MLSA, a digital DNA:DNA hybridization [38] was performed using the Genome-genome distance calculator (GGDC) [39]. A threshold of 70% for digital DNA:DNA hybridization and 1% for difference in percentage guanine-cytosine content were used for determination of species and subspecies boundaries via the Type (Strain) Genome Server [40]. Phylogenetic trees were constructed from the alignment of the whole-genomes and their corresponding 16S rRNA sequences, using a GreedyWithTrimming algorithm on FastME 2.0 [41].

## Results

### Pathogenicity of isolates

**Fig. 1:**
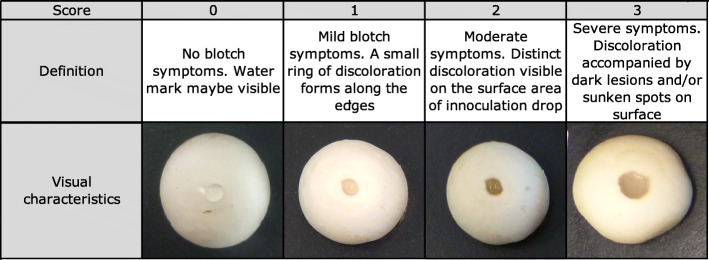
In-vitro pathogenicity assays to quantify the virulence of an isolate when inoculated on fresh mushroom caps. It describes the visual characteristics used to score the blotch symptoms as none, mild, moderate and severe in a pathogenicity assay

**Fig. 2:**
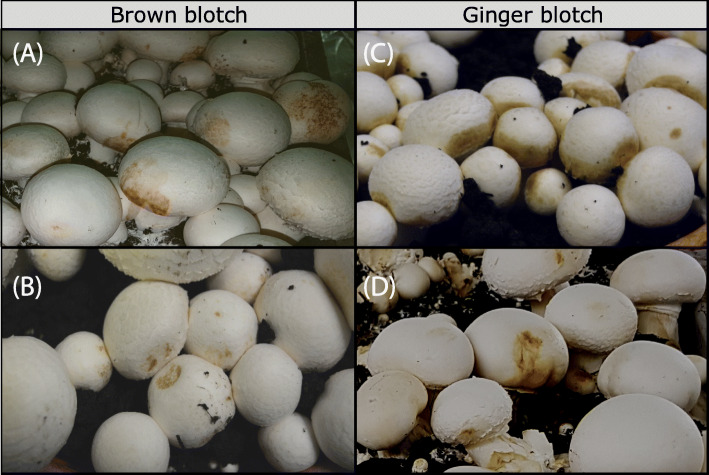
Pathogenicity bioassays in pots to confirm the virulence of isolates when inoculated in the casing soil. Brown blotch symptoms were caused by (**a**) *P. salomonii* (IPO3765) and (**b**) *P. costantinii* (LMG 22119^T^) and ginger blotch symptoms were caused by “*P. gingeri*” isolates (**c**) P8018 and (**d**) IPO3777, in independent pathogenicity bioassays in pots

102 bacterial isolates were tested for their ability to cause bacterial blotch symptoms on fresh mushroom caps (Fig. 1). 6 out of the 17 strains that belonged to international culture collections could cause blotch symptoms. Out of the 85 bacterial isolates recovered from blotched mushroom tissue, 55 isolates caused mild to severe symptoms in the pathogenicity cap test. From this panel, the pathogenicity of 30 bacterial isolates and strains were also validated in the pot test, by inoculation of the pathogen in the casing soil (Fig. 2). The pot test and cap test gave similar results (Additional file 1). The pathogenicity of the isolates is further described in this text with reference to bacterial blotch only.

### Whole genome sequences

In total, whole genome sequences from 113 bacteria were analysed. The consortium of sequenced bacteria contained 85 isolates from symptomatic mushroom tissue and 28 reference strains from international culture collections at LMG (Laboratory of Microbiology, Belgium), ATCC (American Type Culture Collection, United States) and NCPPB (National Collection of Plant Pathogenic Bacteria, United Kingdom). The total number of reads per isolate, averaged across the dataset, were 16,075,321 indicating good sequencing depth, with mean Phred score of 38 which suggests high sequence quality and mean GC content of 63%. Additional file 1 describes the sequence identifiers and their metadata.

### Multi-locus sequence analysis

Eleven taxon-specific sequences that are stable with regard to rapid genetic mutations were selected as barcoding genes from known literature reports [8, 25, 42], namely, *atpD, fusA, glnS, groeL, gyrB, ileS, recA, recN, rpoB, rpoD* and *uvrC* (Table 1). Concatenated sequences of the barcoding genes from 13 reference strains of well-known mushroom-associated *Pseudomonas* species (Table 2) were used for MLSA, such that the variability within the barcoding genes predicts the overall whole genome relatedness [30]. Two of these strains from the *P. fluorescens* group were added to the reference list based on preliminary data exploration. *P. yamanorum* is a psychrotolerant soil bacterium from the *P. fluorescens* group [43], that contains a paralog of the *tolaasin* gene fragment [44]. *Pseudomonas* sp. *NC02* is a recently isolated soil bacterium, which is closely related to *P. yamanorum*.

**Table 1:** List of individual barcoding genes used for predicting whole-genome relatedness in MLSA

Barcoding gene	Description or product	Length variation
*atpD*	ATP synthase subunit beta	0471–1380 bp
*fusA*	Elongation factor G	2106–2148 bp
*glnS*	Glutamine--tRNA ligase	1482–1701 bp
*groeL*	Chaperonin 1	1620–1650 bp
*gyrB*	DNA gyrase subunit B	2379–2424 bp
*ileS*	Isoleucine--tRNA ligase	2106–2832 bp
*recA*	DNA repair protein A	0612–1065 bp
*recN*	DNA repair protein N	1672–1674 bp
*rpoB*	DNA-directed RNA polymerase (subunit B)	4074–4081 bp
*rpoD*	RNA polymerase sigma factor (sigma-70)	0519–1851 bp
*uvrC*	UvrABC system excinuclease (subunit C)	1824–1839 bp

**Table 2:** List of genomes used for extracting the reference sequences of the barcoding genes for MLSA

Reference genomes	Accession (Assembly)
*P. poae* LMG 21465^T^	GCA_001439785.1
*P. protegens* CHAO^T^	GCA_000397205.1
*P. veronii* LMG 17761^T^	GCA_001439695.1
*P. costantinii* LMG 22119^T^	GCA_001870435.1
*P. putida* BIRD 1	GCA_000183645.1
*“P. reactans”* LMG 5329^T^	GCA_000411675.1
*P. agarici* LMG 2112^T^	GCA_900109755.1
*P. tolaasii* LMG 2342^T^	GCA_002813445.1
*“P. gingeri”* LMG 5327^T^	GCA_002895165.1
*P. fluorescens* LMG 1794^T^	GCA_900215245.1
*P. syringae* DC3000	GCA_000007805.1
*P. yamanorum* LMG 27247^T^	GCA_900105735.1
*Pseudomonas* sp. *NC02*	GCA_002874965.1

**Fig. 3:**
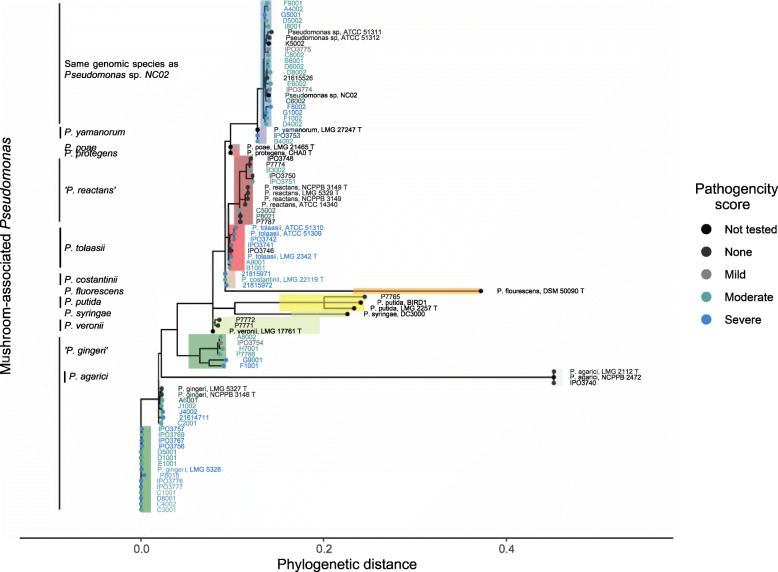
Phylogenetic tree from consensus sequences extracted via Multi-locus Sequence Alignment of barcoding genes. A maximum-likelihood phylogenetic tree in which weak nodes (< 70%) that are not supported by bootstrapping have been collapsed. The colour of the branches indicates the identity of the isolates, based on the mapping of the reference barcoding genes. The colour of the tip labels and tip point indicates the pathogenicity of the isolates on fresh mushroom caps and in pot assays

The largest clusters in the phylogenetic tree are comprised of “*P. gingeri”, Pseudomonas* sp. *NC02 “P. reactans”* and *P. tolaasii,* in decreasing order of size. “*P. gingeri”* and *“P. reactans”* isolates formed multiple clusters within the species (Fig. 3). A few isolates also mapped to barcoding genes from *P. putida, P. agarici, P. veronii, P. costantinii* and *P. yamanorum.* With the exception of *“P. gingeri”*, non-pathogenic isolates were also found in species clusters that contained pathogenic isolates.

None of the isolates mapped to reference strains of *P. poae, P. protegens, P. fluorescens* or *P. syringae.* 15 out of 113 isolates mapped non-specifically, with relatively low percentage identity, to multiple reference strains indicating the presence of other *Pseudomonas* species. 5 isolates did not map to any of the references and could potentially be non-pseudomonads*.* Within species clusters of the phylogenetic tree, the individual isolates had low numbers of substitutions per sequence site, indicating short evolutionary distances within species (Fig. 3).

### Average nucleotide identity

**Fig. 4:**
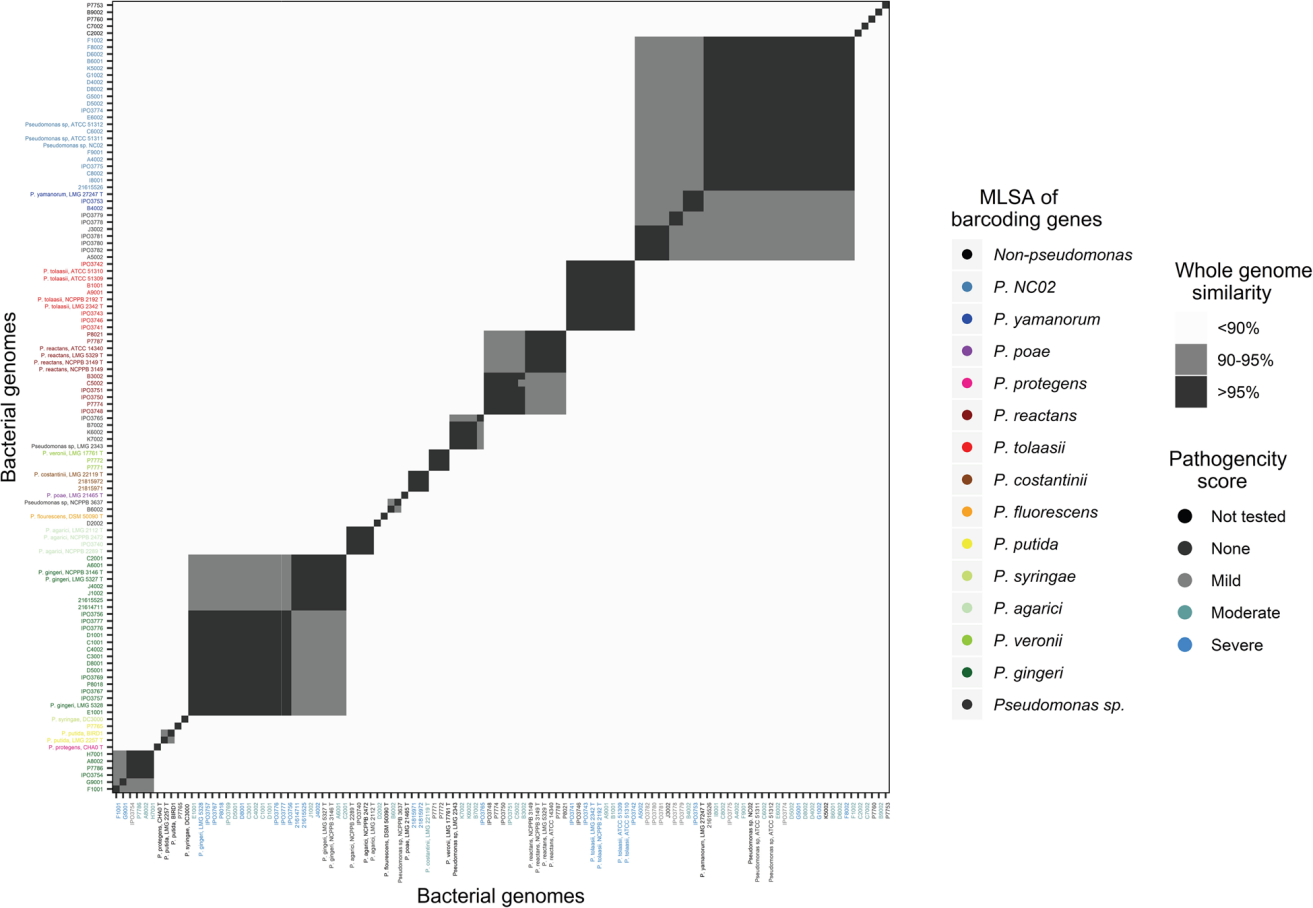
Heatmap from whole-genome similarity values obtained in the Average Nucleotide Identity analysis. Colours of the heatmap indicate the pairwise genome-genome similarity. The labels on the y-axis are coloured according to the identity assumed from the MLSA analyses; the labels on the x-axis are coloured according to the pathogenicity of the isolates on fresh mushroom caps, and in select cases also pot tests

Average nucleotide identity analysis recognized 32 unique bacterial phylotypes associated with the cap tissue of blotched button mushrooms in Western Europe (Fig. 4). These are phylogenetically distinct whole genome sequences differing at species level, based on a < 95% similarity cut-off for delineation. Similar to the MLSA, the largest numbers of blotch pathogens were identified as close relatives of *Pseudomonas* sp. *NC02* or “*P. gingeri”,* although pathogenic isolates of *P. tolaasii*, *P. costantinii*, *“P. reactans”* and *P. yamanorum* were also discovered. Isolates that were unable to cause blotch were primarily identified as *“P. reactans”, P. agarici, P. veronii* and strains belonging to the same species as *Pseudomonas* sp. *NC02*. Within phylotypes, isolates did not cluster according to geographic region, year of outbreak or pathogenicity scores. Twelve unidentified phylotypes consisted of isolates with varying levels of symptom severity on fresh mushroom caps and did not contain any reference or type strains.

### In-silico DNA:DNA hybridization

**Fig. 5:**
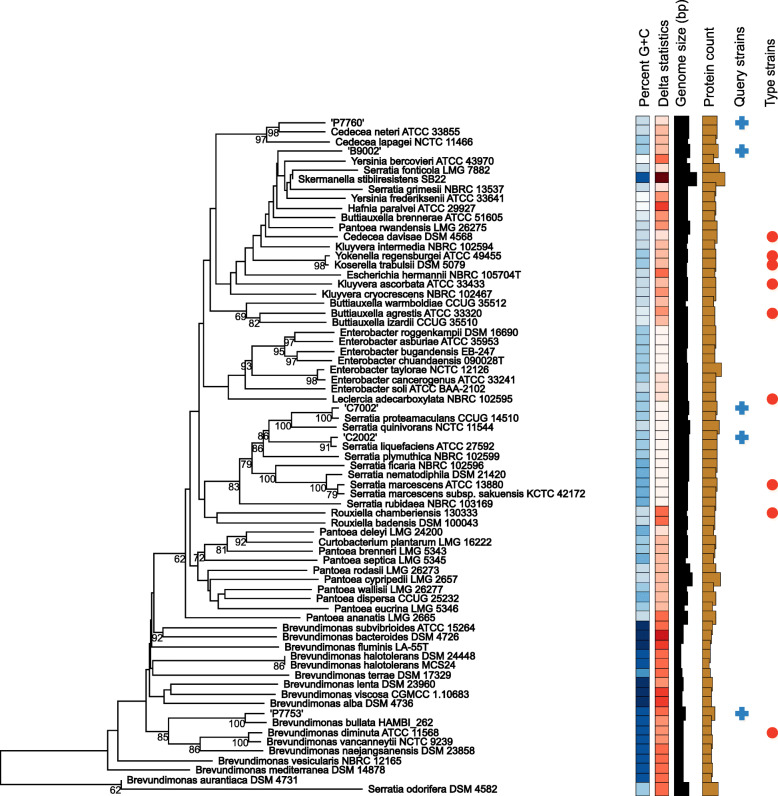
Phylogenetic tree from *in-silico* DNA:DNA hybridization of non-*Pseudomonas* isolates that were unidentified in the MLSA and ANI. A minimum evolution phylogenetic tree rooted at midpoint, in which branch support was inferred from 100 bootstrap replicates each. The genome size, percentage GC content, total protein count and delta statistics are described for 5 query sequences and 9 type strains spanning multiple genera. Low delta statistic values indicate higher accuracy of the phylogeny [45]

Five non-pseudomonad isolates were identified from *in-silico* DDH of the whole genome sequences (Table 3). Two isolates that cause moderate blotch on fresh caps, C2002 (phylotype 15) and C7002 (phylotype 17) were identified as *Serratia liquefaciens* and *Serratia proteamaculans* respectively (Fig. 5). Non-pathogenic isolate P7753 (phylotype 30) was closely related to *Brevundimonas bullata*. Non-pathogenic isolate P7760 (phylotype 31) was closely related to *Cedecea neteri*. Moderate blotch-causing isolate B9002 (phylotype 13) was related to reference genomes of multiple *Pantoea* species.

**Table 3:** *In-silico* DNA:DNA hybridization of unidentified isolates

Query isolate	Phylo-type (ANI)	Reference strain	dDDH value (%)	Confidence intervals (%)	Difference in GC (%) content
Non-*Pseudomonas*					
B9002	13	*Pantoea rodasii* LMG 26273^T^	40.5	37.5–43.5	0.9
C2002	15	*Serratia liquefaciens* LMG 7884^T^	**96.5**	**95.0–97.6**	0.14
C7002	17	*Serratia proteamaculans* NCPPB 245^T^	**93.4**	**91.1–95.1**	0.01
P7753	30	*Brevundimonas bullata* LMG 17157^T^	72	68.5–75.2	0.22
P7760	31	*Cedecea neteri* LMG 7864^T^	63	59.7–66.2	0.55
*Pseudomonas* species					
A5002	4	*P. yamanorum* LMG 27247^T^	69.2	65.8–72.5	0.63
IPO3780	4	*P. yamanorum* LMG 27247^T^	70.3	66.8–73.5	0.73
IPO3781	4	*P. yamanorum* LMG 27247^T^	68.2	64.8–71.4	0.59
IPO3782	4	*P. yamanorum* LMG 27247^T^	70.9	67.4–74.1	0.76
J3002	4	*P. yamanorum* LMG 27247^T^	69.1	65.7–72.4	0.67
B6002	9	Isolate P7548 (phylotype 11)	80.9	77.5–83.9	0.24
P7548	11	*P. fluorescens* DSM 50090^T^	57.3	54.1–60.5	0.61
B7002	12	*P. edaphica* LMG 30152^T^	**88.2**	**85.2–90.6**	0.03
IPO3747	12	*P. edaphica* LMG 30152^T^	**89.9**	**87.1–92.1**	0.1
K6002	12	*P. edaphica* LMG 30152^T^	**89.8**	**87.0–92.0**	0.12
K7002	12	*P. edaphica* LMG 30152^T^	**89.9**	**87.1–92.1**	0.1
B3002	16	*P. reactans* LMG 5329^T^	44.4	41.4–47.4	2.94
D2002	21	*P. fluorescens* DSM 50090^T^	69.4	65.4–73.0	0.15
IPO3765	25	*P. salomonii* LMG 22120^T^	**87.9**	**84.4–90.7**	0.31
IPO3778	26	*P. yamanorum* LMG 27247^T^	64.8	61.5–68.1	0.59
IPO3779	26	*P. yamanorum* LMG 27247^T^	64.8	61.5–68.0	0.6

Only the highest dDDH values from comparing each query strain with multiple reference strains have been reported. Values in bold indicate that the query belongs to the same species as the reference strain, as the confidence intervals meet the 70% threshold for DDH and 1% difference in %GC content. Values that are not highlighted, lie on the border or below the thresholds, and indicate the reference that the query isolate is most closely related to

**Fig. 6:**
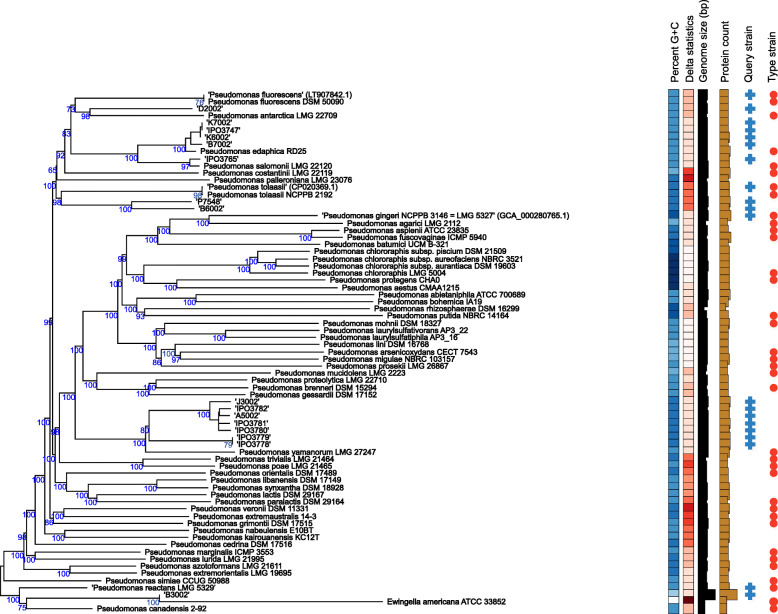
Phylogenetic tree from *in-silico* DNA:DNA hybridization of *Pseudomonas* isolates that were unidentified in the MLSA and ANI. A minimum evolution phylogenetic tree rooted at midpoint, in which branch support was inferred from 100 bootstrap replicates each. The genome size, percentage GC content, total protein count and delta statistics are described alongside the phylogenetic tree for 21 query sequences and 45 type strains from the genus *Pseudomonas*

Among the *Pseudomonas*, three isolates that cause blotch on fresh caps (phylotype 12) belonged to *P. edaphica* and severe blotch-causing isolate IPO3765 (phylotype 25) was identified as *P. salomonii* (Fig. 6). Several *Pseudomonas* spp. did not hybridize sufficiently (> 70%) with any of the type strains or share a similar %GC content (< 1%). Seven of these isolates (phylotypes 4 and 26) were closely related to *P. yamanorum* LMG 27247^T^. Isolates P7548 (phylotype 9) and B6002 (phylotype 11), clustered together as the same genomic species, and were closely related to *P. fluorescens* DSM 50090^T^ (> 60% dDDH). Isolate D2002 (phylotype 21) was also closely related to *P. fluorescens*. Isolate B3002 (phylotype 16) did not map sufficiently with the type strain of “*P. reactans”*, in contrast to the MLSA and ANI results.

### Taxonomic corrections

The taxonomy of several reference strains from culture collections have been corrected based on the combined results from MLSA, ANI and dDDH analyses. ATCC 51312, ATCC 51311, LMG 2343 were formerly identified as *P. tolaasii*, but they share less than 95% genome similarity with *P. tolaasii* genome cluster (phylotype 6). They also do not map to barcoding genes of *P. tolaasii* LMG 2342^T^. Non-pathogenic strains ATCC 51312 and ATCC 51311 were instead identified close relatives of *Pseudomonas* sp. *NC02* (phylotype 2) in the MLSA and ANI, and LMG 2343 (phylotype 12) was identified as *P. edaphica* in the dDDH. Non-pathogenic isolates P7774, P7753 and P7760 were formerly described as *P. veronii, P. syringae* and *P. agarici,* respectively, in internal culture collections. Based on ANI and dDDH results from this study they were re-identified as *“P. reactans”* (phylotype 16), close relative of *Brevundimonas bullata* (phylotype 30) and close relative of *Cedecea neteri* (phylotype 31) respectively.

### Known blotch pathogens

All strains of *P. tolaasii* (phylotype 6) isolated from blotch outbreaks in the Netherlands were found to cause moderate or severe brown blotch symptoms, with the exception of IPO 3746. They also map to barcoding genes of *P. tolaasii* LMG 2342^T^ in the MLSA. Two pathogenic isolates from the United Kingdom, 21,815,971 and 21,815,972 (phylotype 3) that cause severe blotch and pitting in both cap and pot tests, clustered together with the blotch-causing reference strain of *P. costantinii,* LMG 22119^T^ in the MLSA and ANI. Three isolates from the *“P. reactans”* clusters, B3002, C5002, IPO 3751 (phylotypes 9 and 16), were also pathogenic. All 26 isolates of *“P. gingeri”* obtained from blotch outbreaks in the Netherlands, United Kingdom and Belgium were able to cause blotch, with varying symptom severity in the pathogenicity tests performed. All of them mapped to barcoding genes of *“P. gingeri”* LMG 5327^T^. They form 3 MSLA clusters, but 5 distinct ANI clusters (phylotypes 1, 5, 14, 22 and 24), indicating that multiple closely-related species can cause ginger blotch.

### New blotch pathogens

Several isolates presented in this study are reported as blotch-pathogens for the first time. 19 isolates (phylotype 2) with varying severity of blotch symptoms were identified as belonging to the same species as *Pseudomonas* sp. *NC02*. Two blotch-causing isolates (phylotype 10) from the Netherlands were identified as *P. yamanorum*. They were also closely related to the *Pseudomonas* sp. *NC02* isolates. Three pathogenic isolates (phylotype 12) from Dutch blotch outbreaks were identified as *P. edaphica*. A severely pathogenic isolate (IPO3765) from Netherlands was recognized as *P. salomonii*.

Four ANI clusters (phylotypes 4, 9, 21 and 26) contained pathogenic *Pseudomonas* isolates without a reference or type strain. Phylotype 4 contained only one severe blotch-causing isolate, A5002. Phylotypes 9 and 21 contained only a single isolate each, both of which (B6002 and D2002) cause brown blotch symptoms. Phylotype 26 contained two unidentified isolates, IPO 3778 and IPO 3779 that cause mild-blotch on fresh caps.

### Unidentified *Pseudomonas* species

10 isolates demonstrating a range of blotch severity, from non-pathogenic to severe symptoms, remained unidentified. They mapped to barcoding genes from multiple reference strains in the MLSA, formed 7 distinct clusters (phylotypes 4, 9, 11, 21, 26, 30 and 31) in the ANI analysis (Fig. 4), and were closely but insufficiently (< 70%) related to any other species in the dDDH (Table 3). Their contigs mapped in-specifically to multiple reference strains, when compared via BLAST against the NCBI database. Contamination of these isolates during laboratory work was ruled out by confirming the presence of single-copies of the barcoding genes, and low genome duplication in the range of 1.50–2.84% (Additional file 2). These could potentially be new species that have yet to be discovered.

### Non-pathogenic *Pseudomonas*

Five non-pathogenic isolates, P7787, P8021, P774, IPO 3748 and IPO3750, mapped to barcoding genes from “*P. reactans”* LMG 5329^T^ in the MLSA, but were split over phylotype 8 and 16 in the ANI analysis. All isolates from phylotype 19 did not cause blotch and mapped to barcoding genes of *P. agarici* LMG 2112^T^, better known as the causative agent of drippy gill. Fluorescent *Pseudomonas* spp. such as *P. putida* (phylotype 23, 29, 30), *P. veronii* (phylotype 27) and *P. poae* (phylotype 20) that are usually considered as beneficial organisms, were also confirmed to be non-pathogenic.

## Discussion

### Genetic diversity of blotch-causing *Pseudomonas*

The established phylogeny of bacterial isolates associated to symptomatic cap tissue is largely consistent between the MLSA, ANI and dDDH analyses. This substantiates the conclusions made about the identity of blotch pathogens, and the related taxonomic corrections. *“P. gingeri”, P. tolaasii, “P. reactans” and P. costantinii* are known causative agents of bacterial blotch [4, 11, 17, 24]. In addition, several novel blotch pathogens were identified in these analyses, namely *P. yamanorum, P. edaphica, P. salomoni* and strains clustering together with *Pseudomonas* sp. *NC02,* as the same genomic species*.*

*P. yamanorum* is a recently described psychrotolerant member of the *P. fluorescens* group that was found in Argentinian soil under cold and humid climatic conditions [43]. In a recent study, the tolaasin gene fragment of a few brown blotch-causing isolates in Turkey were also found to share homology with that of *P. yamanorum* [44]. *Pseudomonas* sp. *NC02* is a recently isolated soil bacterium about which not much is known [46]. *P. edaphica* is another newly identified fluorescent *Pseudomonas* that was isolated from rhizosphere soil, and is closely related to *P. brenneri* [47]. *P. salomonii* was first characterized as a pathogen of ‘Café au lait’ disease on garlic [48], and was later also recovered from ready-to-eat vegetables [49]. This is the first report of the blotch-causing ability of this pathogen on mushrooms. Its pathogenicity in the cap test was also verified by pot tests.

*“P. gingeri”* and *“P. reactans”* are both invalidly named species known to cause bacterial blotch [17, 18, 50]. In this study, all isolates previously classified as *“P. gingeri”* and *“P. reactans”* map to barcoding genes of their respective reference strain, although they form multiple distinct clusters in the MLSA as observed from the phylogenetic tree (Fig. 3). They also split into multiple phylotypes in the ANI analysis instead of clustering together (Fig. 4). Delineation of these phylotypes on a genome level (< 95% similarity) despite mapping to barcoding references from the same species, perhaps indicates the existence of a species complex with multiple taxonomically-related species, as opposed to other blotch pathogens which cluster as single phylotypes.

On the basis of genomic and phylogenetic analyses such as MLSA, ANI and dDDH, this study also reports four blotch-causing *Pseudomonas* spp. (phylotypes 4, 9, 21 and 26) which do not belong to any of the existing species within the genus *Pseudomonas*. These are as yet unidentified, and could potentially be new species. The taxonomic assignment of these novel blotch-causing phylotypes as new species requires a polyphasic approach involving additional phenotypic and chemotaxonomic data such as fatty acid methyl esters, polar lipids and respiratory quinones, etc. [51].

### Pathogenicity and phylogeny

Koch’s postulates were evaluated to identify causative agents of bacterial blotch [52]. Bacteria were isolated from symptomatic mushroom tissue and successfully grown to pure culture. In every case, blotch symptoms could be reproduced when isolates were inoculated on mushroom caps. The 4th postulate of Koch, i.e. re-isolation of pathogen from the experimental host and its identification as originally inoculated pathogen, was not fulfilled. The mushroom cap surfaces also harbour endemic *Pseudomonas* spp. that are closely related to the isolates, and have similar characteristics and colony morphology [53, 54]. Due to lack of diagnostic methods, the inoculated isolates cannot be easily differentiated from the endemic microflora. However, the disease developement can be attributed to the inoculated pathogens with certainty, due to the presence of multiple mock-inoculated negative controls which did not develop any blotch symptoms.

24, 8, 39 and 28 isolates were confirmed to cause non-pathogenic, mild, moderate and severe blotch symptoms respectively, on fresh mushroom caps in an in-vitro pathogenicity test. Bacterial isolates did not cluster together according to their pathogenicity scores in the MLSA or ANI analysis. Often blotch-causing and non-pathogenic isolates were part of the same phylotype or branch (Figs. 3 and 4). This elaborates the need for a pan-genomic analysis to identify the pathogenicity determinants of bacterial blotch diseases. Fluorescent *Pseudomonas* share a rather small core genome of 1344-protein coding genes [55]. The size and diversity of the pan-genome is thus determined by secondary metabolites biosynthesis clusters that can be strain specific and responsible for pathogenicity [56]. Pan-genomic elements shared exclusively by pathogenic isolates can be ideal targets for the further development of DNA-based diagnostic tools.

### Regional diversity of blotch-causing *Pseudomonas*

Within species, bacterial isolates did not cluster together according to their geographic region or year of outbreak. The two Belgian isolates (IPO 3781 and IPO 3782), both caused mild ginger blotch and clustered together with those of the Netherlands. Two Dutch isolates of “*P. gingeri*” (IPO 3757 and IPO 3756) that caused severe ginger blotch, clustered differently from each other in phylotypes 14 and 24 respectively. The phylogenetic distribution of the common pathogens independently from their region of outbreak indicates that Western Europe shares a well-mixed pathogen pool.

However, a few potentially region-specific blotch pathogens were also recovered in this study. *P. yamanorum, P. edaphica* and *P. salomonii* isolates have so far only been found in the Netherlands. *P. costantinii* isolates in this study, 21,815,971 and 21,815,972, which cause brown blotch and pitting, have been found only in the United Kingdom, although other blotch-causing *P. costantinii* have been reported in Finland too [57]. It is also important to consider that very few isolates from these species were found in this study. This regional exclusivity could also be an artefact of low sampling due to the low presence of these species.

### Blotch causing non-pseudomonads

Two *Serratia* species, *S. liquefaciens, (*C2002, phylotype 15) and *S. proteamaculans, (*C7002, phylotype 17) were recovered from blotched caps in this study. *Serratia* species are present in casing soil, compost, and on mushroom caps [53, 58–60]. *Serratia liquefaciens* is a known agent of yellow blotch on the caps of *A. bisporus* [61]. They cause browning of the *A. bisporus* sporophore [62] by secreting chitinolytic enzymes and chitin-binding proteins [63]. They also produce a surfactant, serwettin, which enables the bacterium to colonise fresh areas of the mushroom cap, as tolaasin does [64, 65]. *P. fluorescens* also uses a similar mechanisms to spread on broccoli heads [66].

A moderately pathogenic *Pantoea* isolate (B9002, phylotype 13) was also identified in this study. *Pantoea* species have been previously isolated from caps of *A. bisporus* [60], and are frequently recovered from soil and water environments too [67, 68]. This gram negative bacterium is an agent of soft rot disease on the stipes and pileus of *Pleurotus eryngii* [69], although it has not yet been reported to be a pathogen of *A. bisporus*. *Pantoea* species from waste mushroom beds are also used for production of plant auxin, indole-acetic acid, for phosphate solubilization [70].

A non-pathogenic *Cedecea* isolate (P7760, phylotype 31), closely related to *C. neteri,* was also found on a blotched mushroom cap*. Cedecea* were one of the most common genera recovered from blotched tissue of *A. brazilensis* [71] and is often present on *A. bisporus* also [72]. Although *Cedecea neteri* is an agent of soft rot in *Pholiota nameko* [73] and yellow sticky disease in *Flammulina velutipes* [74]*,* only *Cedecea davisae* strains have been reported to cause blotch in *A. bisporus* [61].

### Beneficial bacteria on symptomatic cap tissue

Non-pathogenic and potentially beneficial bacteria were also recovered from cap tissue of blotched mushrooms. *P. putida* (P7771, P7772, phylotype 27) and *P. veronii* (P7765, phylotype 32) isolates were confirmed to be non-pathogenic on fresh mushroom caps. *P. putida, P. poae* and *P. veronii* are known to stimulate primordia formation of *A. bisporus* by the removal of volatile C8 compounds [75, 76]. The relative abundance of these beneficial bacteria on blotched caps is yet to be explored, in relation to disease expression by blotch causing bacteria. *Brevundimonas* isolate, P7753 (phylotype 30), closely related to *B. bullata* was also discovered on symptomatic cap tissue. During the cultivation cycle, increased *Brevundimonas populations* have been observed in the casing soils from *A. bisporus* and *P. ostreatus* farms [58, 77, 78], and *Brevundimonas* sp. can be thus expected to also colonize mushroom caps. It has also been reported as a symbiont on the apothecia of the cup fungus, *Scutellinia scutellata* [79].

## Conclusion

This study provides a detailed overview of the regional, genetic and phenotypic diversity of bacterial blotch pathogens in Western Europe. It describes the presence of known pathogens inhabiting blotched mushroom caps: *“P. gingeri”, P. tolaasii, P. costantinii* and *“P. reactans”*. It also reports seven new pathogens of bacterial blotch: four *Pseudomonas species*, *P. yamanorum, P. edaphica, P. salomonii* and strains belonging to the same species as *Pseudomonas* sp. *NC02*, and three non-pseudomonads, *S. liquefaciens, S. proteamaculans* and a *Pantoea* sp. Their epidemiology and aetiology deserve further attention. This molecular investigation of bacterial species that colonize blotched mushroom caps allows further study of the mechanisms and microbial ecology of symptomatic disease expression on the *A. bisporus* sporophore. It also highlights the need for development of diagnostic assays against these newly discovered pathogens. Several new blotch-causing phylotypes discovered in this study are as yet unidentified and require additional chemotaxic data for their taxonomic assignment as new species.

## Supplementary information

**Additional file 1.** Isolates and reference strains that were sequenced, with metadata on initial identity, pathogenicity, source, region of outbreak, year of outbreak, MLSA, ANI and dDDH.

**Additional file 2 **Barcoding genes and genome duplication in *Pseudomonas* isolates that map in specifically and remain unidentified. It describes the number of copies of nine barcoding genes found in the assembled genome, and the overall percentage of genome duplication in the assembly.

## Data Availability

The datasets generated and/or analysed during the current study are available in the NCBI repository under BioProject number PRJNA607442.
